# Association of Interleukin-10 Polymorphisms with Schizophrenia: A Meta-Analysis

**DOI:** 10.1371/journal.pone.0090407

**Published:** 2014-03-06

**Authors:** Lei Gao, Zhao Li, Suhua Chang, Jing Wang

**Affiliations:** 1 Key Laboratory of Mental Health, Institute of Psychology, Chinese Academy of Sciences, Beijing, China; 2 University of Chinese Academy of Sciences, Beijing, China; Rikagaku Kenkyūsho Brain Science Institute, Japan

## Abstract

**Background:**

The involvement of cytokines in schizophrenia (SZ) has been proposed in recent years and various studies have accumulated convergent lines of evidence. Among which, the role of interleukin-10 (*IL-10*) in SZ has been explored in a number of studies by investigating association of single nucleotide polymorphisms (SNPs) and susceptibility of SZ. However, the results are inconsistent since its power is limited by the individual sample size. To evaluate the overall effect between them, we conducted a meta-analysis by combining all available studies.

**Methods:**

Studies were searched from the database of PubMed, PsycINFO and ISI web of Knowledge up to Nov 2013. The meta-analysis was conducted based on statement of preferred reporting items for systematic reviews and meta-analyses (PRISMA).

**Results:**

Eleven studies including 6399 subjects (3129 cases and 3270 controls) were available for the meta-analysis. Among three investigated SNPs, rs1800872 was observed to be significantly associated with risk of SZ (AA vs. AC+CC, Pooled OR = 1.351, *P*-value  = 2.06E-04). Meanwhile, among six haplotypes of rs1800896 - rs1800871 - rs1800872, significant associations were observed in haplotype A-C-A (Pooled OR = 1.762, *P*-value  = 2.00E-03) and G-C-C (Pooled OR = 0.649, *P*-value  = 2.00E-03) for Asians. These results were still significant after adjusting for multiple comparisons.

**Conclusions:**

This meta-analysis demonstrated an SNP and two haplotypes of *IL-10* significantly associated with SZ, suggesting that *IL-10* might be a risk factor of SZ.

## Introduction

Schizophrenia (SZ) is a complex psychiatric disorder which affects approximately 1% of the population worldwide [1]. It has been demonstrated that both genetic and environmental factors contribute to SZ, but the etiology is still unclear [2]. To elucidate the pathogenic mechanism of SZ, multiple hypotheses have been proposed such as neurodevelopmental hypothesis [3], dopamine hypothesis [4], glutamate hypothesis [5] and cytokine imbalance hypothesis [6]. Among these theories, the cytokine imbalance hypothesis, which implies that imbalance of cytokines represents a key mechanism involved in the precipitation of schizophrenia-related pathology, is drawing growing attention of researchers, during the past two decades [7]. Cytokines, as key signaling molecules in inflammation, their regulatory effect extends beyond the inflammatory system, impacting also on neurotransmitter metabolism, neurogenesis and the neuroendocrine system [8]–[9].

During these decades, researches from different areas have provided convergent lines of evidence for the involvement of cytokines in SZ. Comprehensive meta-analyses of clinical studies showed that compared with healthy controls, patients with SZ had significant inflammatory cytokine alterations [10]. Furthermore, studies on animal models also indicated that cytokines could induce schizophrenia-like behavior in animals [11]–[12]. Besides, clinical studies demonstrated that antipsychotic drugs could produce anti-inflammatory effects by altering some cytokine levels in SZ patients [13]. Meanwhile, it was also reported that anti-inflammatory drugs could improve the symptoms of SZ patients [14].

Among those investigated cytokines, the involvement of interleukin-10 (*IL-10*) in SZ has been supported by a variety of evidence. *IL-10* was an anti-inflammatory cytokine that regulates the inflammatory response, by inhibiting pro-inflammatory cytokine production [15]. A previous study demonstrated that the genetically enforced expression of *IL-10* by macrophages attenuates behavioral abnormalities in a mouse model [16]. Furthermore, comprehensive meta-analyses of clinical studies demonstrated that blood *IL-10* levels were significantly decreased in acutely relapsed inpatients of SZ [17]. Meanwhile, it was also observed that blood *IL-10* levels were associated with severity of symptoms in SZ patients [18]. Besides, there was also studies reporting that atypical antipsychotics could up-regulate *IL-10* level [19].

Both clinical and epidemiological evidence has supported the cytokine imbalance hypothesis. With the emergence of the evidence, abundant genetic researches have been conducted to explore the genetic basis of this hypothesis. Among these cytokines, the role of interleukin-10 (*IL-10*) in SZ has been explored in a number of studies by investigating association of single nucleotide polymorphisms (SNPs) and susceptibility of SZ [20]–[29]. However, results of individual studies were inconsistent and might not be powerful enough due to the limited sample size. To evaluate the overall effect of *IL-10* polymorphisms on SZ, a meta-analysis was conducted in the present study by pooling all available data together.

## Materials and Methods

The meta-analysis was conducted according to PRISMA statement (Preferred reporting items for systematic reviews and meta-analyses) [31], including search strategy, selection criteria, data extraction and data analysis.

### Search Strategy

The database of PubMed, PsycINFO and ISI web of Knowledge were searched up to November 2013 using the following search terms: (“interleukin 10” OR “interleukin-10” OR “IL10” OR “IL 10” OR “IL-10”) AND “Schizophrenia”. Publication date and publication language were not restricted in our search. Reference lists and supplemental materials were also examined manually to further identify potentially relevant studies. Meanwhile, published genome-wide association studies (GWASs) about schizophrenia were also examined. Furthermore, we also contacted the authors to ask for original genotype data and related information if insufficient data were provided. If overlapped samples were used in different studies, we excluded overlapping samples or keep the study with the largest sample size.

### Selection Criteria

Studies aiming to examine the association between *IL-10* polymorphisms and susceptibility of SZ were included. Moreover, studies had to fulfill all of the following criteria: 1) a case-control design comparing patients with SZ to controls without mental disorders; 2) patients were diagnosed with well-validated diagnostic criteria (e.g. The Diagnostic and Statistical Manual of Mental Disorders); 3) controls were free of autoimmune or inflammatory diseases; 4) original data of genotype frequencies were published or provided by the authors. Studies were excluded if one of the following existed: 1) studies used family-based or cohort design; 2) samples were cases only; 3) genotype frequencies were neither published nor provided; 4) information is still insufficient for the meta-analysis even after requesting from authors.

### Data extraction

All data were extracted independently by two authors according to the inclusion criteria listed above. Disagreements were resolved by discussion between the two authors. The following characteristics were collected from each study: the first author, publication year, geographic region, ethnicity, diagnostic criteria, gender component, sample size, age of cases, age of controls, SNPs/haplotypes investigated, and distribution of genotypes among cases and controls for each involved SNP/haplotype.

### Data analysis

The statistical analysis was conducted using STATA 11.0 (Stata Corp LP, College Station, TX, United States). The strength of association was expressed as pooled odds ratio (OR) along with the corresponding 95% confidence interval (CI), which were estimated for each study in a random-effects model or in a fixed-effects model. If there was a significant heterogeneity (*P*-value <0.1), a random-effects model (the DerSimonian and Laird method) was selected to pool the data. Otherwise, a fixed-effects model (the Mantel-Haenszel method) was selected to pool the data.

As suggested in previous studies [32]–[33], for each polymorphism, pooled ORs were calculated under the following genetic models: additive model (allele a vs. allele A), dominant model (a/a+A/a vs. A/A), recessive model (a/a vs. A/a+A/A), in which “a” represented the minor allele and “A” represented the major allele. The significance of pooled ORs was determined by Z-test and *P*-value <0.05 was considered as statistically significant. Subgroup analyses were also conducted to assess any moderating effects of ethnicity (Caucasian and Asian) on odds ratios derived from each study if significant heterogeneity was observed in the meta-analysis. Moreover, corrections for multiple comparisons were conducted by the Bonferroni method [34].

Hardy-Weinberg Equilibrium (HWE) in the controls was tested by the chi-square test for goodness of fit, using a previous meta-analysis as reference [35], and a *P*-value <0.01 was considered as significant deviation from HWE. As deviations from HWE in control subjects may bias the estimates of genetic effects in a meta-analysis [36], sensitivity analysis was conducted to examine such influence by removing studies with significant deviation from HWE in control subjects and recalculating the pooled OR and 95% CI.

Heterogeneity among studies was examined with the χ^2^ -based Q testing and I^2^ statistics [37]. *P*-value <0.1 was considered significant for the χ^2^-based Q testing and I^2^ was interpreted as the proportion of total variation contributed by between-study variation [37]. Publication bias was examined with funnel plots and Egger's tests [38]. If there is evidence of publication bias, the funnel plot is noticeably asymmetric. For the Egger's tests, the significance level was set at 0.05.

## Results

### Study Characteristics

**Figure 1:**
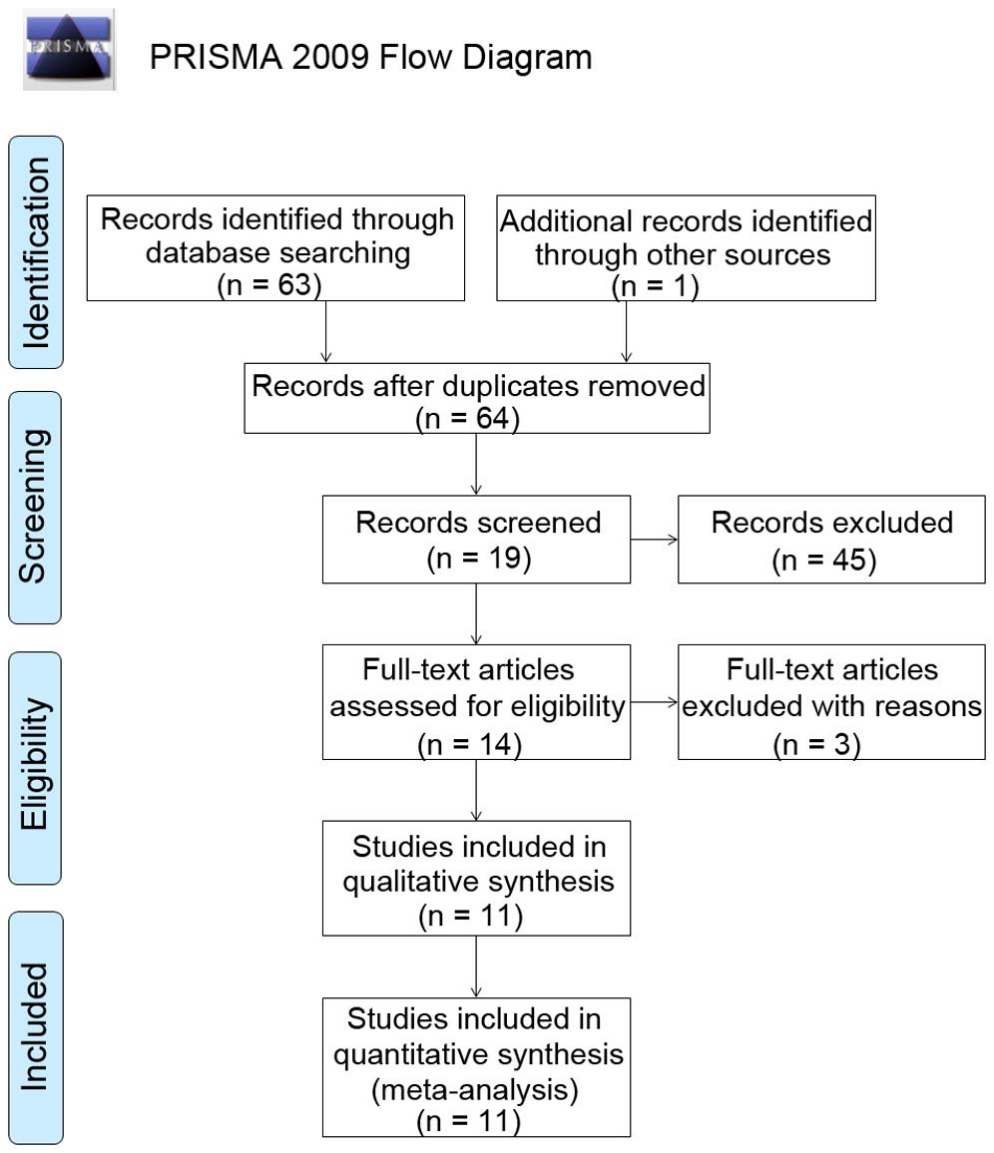
Flow chart of study selection.

A total of 63 papers were obtained with the initial search of databases. After screening, ten studies fulfilled the inclusion criteria, from which genotype data of three SNPs of *IL-10* were obtained [20]–[29]. Furthermore, one dataset of genotype frequencies of one SNP (rs1800872) were also acquired from a genome-wide association study of schizophrenia [30]. Combining data of candidate gene association study with GWAS data, eleven studies with a total of 6399 participants (3129 cases and 3270 controls) were available for this meta-analysis (shown in Table 1). The qualities of these studies were considered accessible for the meta-analysis. The flow chart of selection of studies and reasons for exclusion are presented in Figure 1. Data of three *IL-10* SNPs (rs1800896, rs1800871 and rs1800872) and six haplotypes of rs1800896-rs1800871-rs1800872 were meta-analyzed (shown in Table 2). Characteristics of studies and genotype frequencies were presented in Tables 1 and 2 respectively.

**Table 1:** Characteristics of the included studies.

Study					Cases			Controls			
	Diagnostic	Country				Male	Age		Male	Age	Ref
	criteria	/Area	Ethnicity	Investigated SNPs	N	(%)	(year)	N	(%)	(year)	
Bocchio(2002)	DSM-IV	Italy	Caucasian	rs1800896, rs1800871, rs1800872	106	NA	NA	143	NA	NA	[20]
Yu(2004)	DSM-IIIR	China	Asian	rs1800896, rs1800871, rs1800872	341	54	42±16	334	52	42±16	[21]
Shirts(2006)	DSM-IV	USA	Caucasian	rs1800872	471	65	38±10	453	52	NA	[22]
Peng(2008)	DSM-IV	Taiwan	Asian	rs1800896, rs1800871, rs1800872	659	70	36±11	411	43	45±14	[23]
Ozbey(2009)	DSM-IV	Turkey	Asian	rs1800896, rs1800871, rs1800872	171	45	38±11	168	45	36±15	[24]
Almoguera(2011)	DSM-IV	Spain	Caucasian	rs1800896, rs1800871, rs1800872	241	63	NA	435	46	NA	[25]
PGC(CATIE)(2011)	DSM-IV	USA	Caucasian	rs1800872	395	NA	NA	391	NA	NA	[30]
Jun(2003)	DSM-IV	Korea	Asian	rs1800896	233	41	32	181	46	32	[26]
Jun(2002)	DSM-IV	Korea	Asian	rs1800871	141	NA	NA	146	NA	NA	[27]
Paul-Samojedny(2010)	DSM-IV	Poland	Caucasian	rs1800896	96	38	45±12	120	33	39±10	[28]
Lung(2011)	DSM-IV	Taiwan	Asian	rs1800896, rs1800871, rs1800872	233^a^	78	NA	433	44	45±14	[29]

PGC(CATIE): data from samples in Clinical Antipsychotic Trials of Intervention Effectiveness of Psychiatric Genomics Consortium; SNPs: single nucleotide polymorphisms; DSM: diagnosis and statistical manual of mental health disorders; NA:not available; ^a^Cases after excluding overlapping samples with study [23].

**Table 2:** Genotype frequencies of investigated SNPs/haplotypes.

SNP/Haplotype		No. of	Genotype frequencies of SNPs/haplotypes				
	Study	case/control	Genotype	Case	Control	HWE	Ref
rs1800871	Jun(2002)	141/146	CC/CT/TT	0.092/0.44/0.468	0.089/0.425/0.486	0.919	[27]
	Yu(2004)	341/334		0.170/0.372/0.457	0.183/0.392/0.425	0.002	[21]
	Peng(2008)	659/411		0.111/0.358/0.531	0.100/0.362/0.538	0.031	[23]
	Ozbey(2009)	171/173		0.170/0.374/0.456	0.183/0.391/0.426	0.027	[24]
rs1800872	Bocchio(2002)	106/143	AA/AC/CC	0.104/0.349/0.547	0.077/0.455/0.469	0.378	[20]
	Yu(2004)	341/334		0.302/0.513/0.185	0.213/0.577/0.210	0.004	[21]
	Shirts(2006)	471/453		0.087/0.321/0.592	0.046/0.416/0.538	0.057	[22]
	Peng(2008)	659/411		0.51/0.417/0.073	0.496/0.404/0.100	0.400	[23]
	Ozbey(2009)	171/168		0.304/0.515/0.181	0.214/0.589/0.196	0.064	[24]
	Almoguera(2011)	241/244		0.091/0.365/0.544	0.066/0.367/0.567	0.719	[25]
	PGC(CATIE)(2011)	395/391		0.068/0.359/0.572	0.046/0.332/0.621	0.908	[30]
rs1800896	Bocchio(2002)	106/143	GG/GA/AA	0.217/0.368/0.415	0.084/0.406/0.510	0.92	[20]
	Jun(2003)	233/236		0.000/0.180/0.820	0.017/0.133/0.851	0.086	[26]
	Yu(2004)	341/334		0.000/0.141/0.859	0.003/0.141/0.856	0.521	[21]
	Peng(2008)	659/411		0.005/0.060/0.935	0.002/0.112/0.886	0.719	[23]
	Ozbey(2009)	171/168		0.000/0.140/0.860	0.006/0.143/0.851	0.995	[24]
	Paul-Samojedny(2010)	96/120		0.302/0.635/0.063	0.133/0.742/0.125	<0.001	[28]
	Almoguera(2011)	241/278		0.141/0.415/0.444	0.163/0.506/0.331	0.395	[25]
	Yu(2004)	341/334	A-C-A	0.063	0.048	NA	[21]
	Peng(2008)	659/411		0.032	0.009		[23]
	Ozbey(2009)	171/168		0.015	0.012		[24]
	Lung(2011)	275/433		0.013	0.009		[29]
	Bocchio(2002)	106/143	A-C-C	0.321	0.413	NA	[20]
	Yu(2004)	341/334		0.246	0.259		[21]
	Peng(2008)	659/411		0.229	0.232		[23]
	Ozbey(2009)	171/168		0.061	0.065		[24]
	Almoguera(2011)	269/381		0.335	0.341		[25]
	Bocchio(2002)	106/143	A-T-A	0.278	0.304	NA	[20]
	Yu(2004)	341/334		0.475	0.454		[21]
rs1800896-	Peng(2008)	659/411		0.688	0.685		[23]
rs1800871-rs1800872	Ozbey(2009)	171/168		0.120	0.113		[24]
	Almoguera(2011)	269/381		0.247	0.248		[25]
	Yu(2004)	341/334	A-T-C	0.145	0.165	NA	[21]
	Peng(2008)	659/411		0.015	0.016		[23]
	Ozbey(2009)	171/168		0.035	0.042		[24]
	Bocchio(2002)	106/143	G-C-C	0.401	0.283	NA	[20]
	Yu(2004)	341/334		0.048	0.060		[21]
	Peng(2008)	659/411		0.034	0.054		[23]
	Ozbey(2009)	171/168		0.012	0.015		[24]
	Almoguera(2011)	269/381		0.314	0.411		[25]
	Lung(2011)	275/433		0.027	0.054		[29]
	Yu(2004)	341/334	G-T-A	0.021	0.000	NA	[21]
	Peng(2008)	659/411		0.002	0.005		[23]
	Ozbey(2009)	171/168		0.006	0.000		[24]

HWE: Hardy-Weinberg equilibrium; NA: not applicable.

### Meta-analysis of SNPs and association with schizophrenia

**Figure 2:**
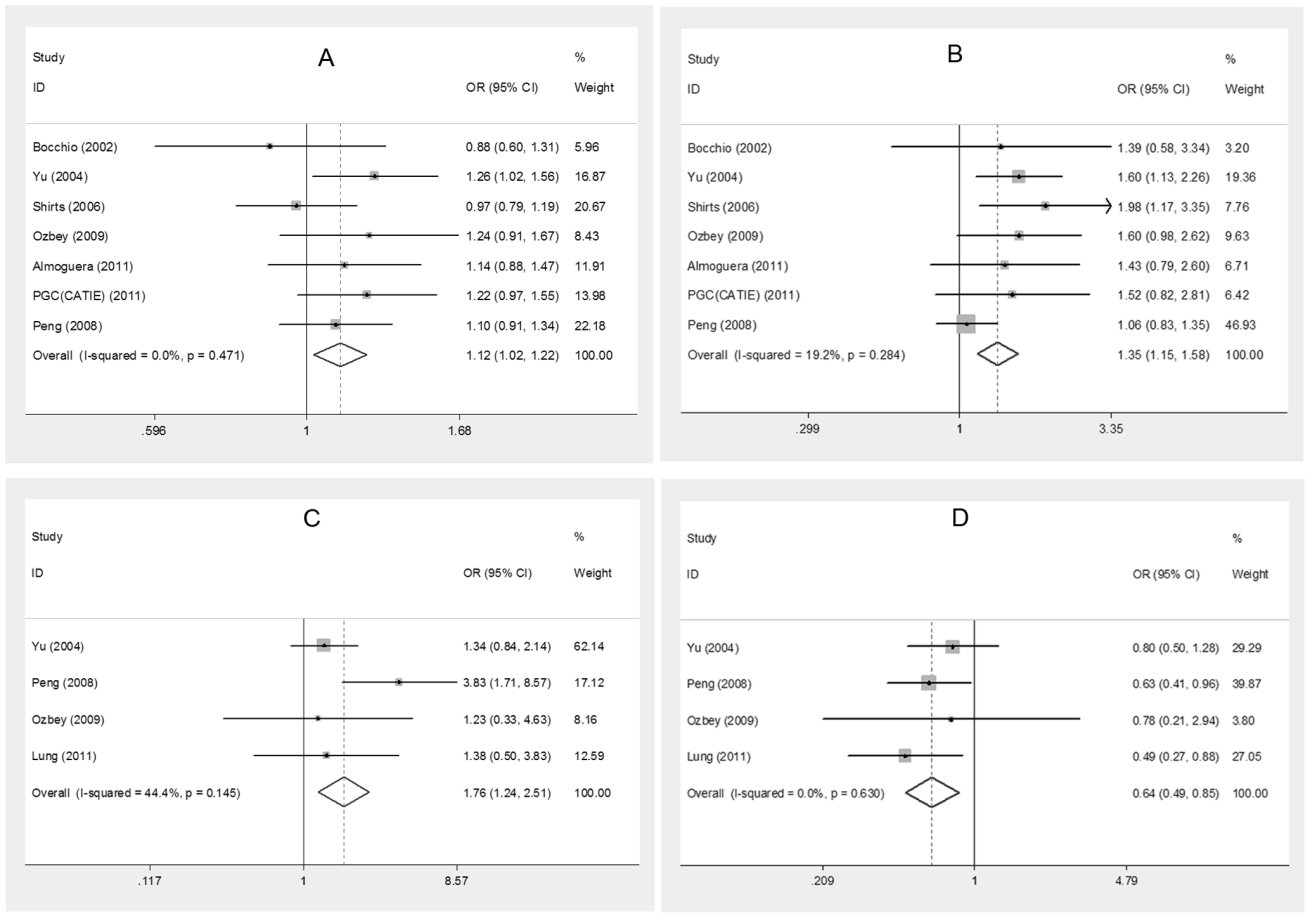
Forest plots of pooled odds ratios in meta-analysis. **A**. Allele A vs. allele C (rs1800872). **B**. AA vs. AC+CC (rs1800872). **C**. Haplotype A-C-A (Asian samples). **D**. Haplotype G-C-C (Asian samples).

The meta-analyses between three SNPs (rs1800896, rs1800871 and rs1800872) and SZ have been conducted, and significant associations were observed only in rs1800872 (allele A vs. allele C, OR = 1.12, *P*-value  = 0.014; A/A vs. C/A+C/C, OR = 1.351, *P*-value  = 2.06E-04). The result of recessive model (A/A vs. C/A+C/C) still remained significant even after correcting for multiple comparison with a *P*-value of 1.86E-03. No significant associations were observed in other SNPs (See Table 3 and Figure 2).

**Table 3:** Results of meta-analysis.

SNP/Haplotype	Genetic model	Meta-analysis		Heterogeneity		Bias
		Pooled OR(95% CI)	P-value	I^2^	P-value	P-value
rs1800096	allele G vs. allele A	1.022(0.758 – 1.377)	0.888	74.5%	0.001	0.566
		1.24(0.698 – 2.203)	0.462^a^	89.2%	0.000	0.049
		0.868(0.667 – 1.128)	0.289^b^	15.0%	0.317	0.863
	GG+GA vs. AA	0.953(0.688 – 1.32)	0.772	64.8%	0.009	0.112
		1.147(0.537 – 2.449)	0.723^a^	82.7%	0.003	0.322
		0.886(0.625 – 1.254)	0.494^b^	45.4%	0.139	0.964
	GG vs. GA+AA	1.351(0.621 – 2.942)	0.448	66.1%	0.007	0.747
		1.858(0.746 – 4.63)	0.183^a^	84.7%	0.001	0.079
		0.503(0.119 – 2.127)	0.350^b^	0.0%	0.447	0.187
rs1800871	allele C vs. allele T	0.98(0.867 – 1.109)	0.752	0.0%	0.735	0.828
	CC+CT vs. TT	0.967(0.822 – 1.139)	0.688	0.0%	0.797	0.837
	CC vs. CT+TT	0.997(0.785 – 1.267)	0.980	0.0%	0.888	0.917
rs1800872	allele A vs. allele C	**1.12(1.023 – 1.225)**	**0.014**	0.0%	0.471	0.744
	AA+AC vs. CC	1.016(0.900 – 1.147)	0.796	21.6%	0.265	0.997
	AA vs. AC+CC	**1.351(1.153 – 1.584)**	**2.06E-04**	19.2%	0.284	0.12
rs1800896- rs1800871-rs1800872	G-C-C vs. non G-C-C	0.784(0.533 – 1.153)	0.216	77.1%	0.001	0.927
		1.042(0.412 – 2.636)	0.930^a^	94.3%	0.000	NA
		**0.649(0.494 – 0.853)**	**2.00E-03^b^**	0.0%	0.759	0.561
	A-C-A vs. non A-C-A	**1.762(1.238 – 2.507)**	**2.00E-03^b^**	44.4%	0.145	0.778
	A-T-C vs. non A-T-C	0.871(0.674–1.126)	0.292^b^	0.0%	0.957	0.725
	G-T-A vs. non G-T-A	3.037(0.132–70.084)	0.488^b^	79.0%	0.008	0.263
	A-C-C vs. non A-C-C	0.927(0.822 – 1.046)	0.220	0.0%	0.507	0.36
	A-T-A vs. non A-T-A	1.023(0.912–1.147)	0.699	0.0%	0.914	0.528

a studies with Caucasian samples were included; ^b^studies with Asian samples were included; NA: not available; OR: Odds ratio; CI: confidence interval; significant results of pooled ORs are presented in bold.

### Meta-analysis of haplotypes and association with schizophrenia

Among six haplotypes of rs1800896-s1800871-rs1800872 (A-C-A, A-C-C, A-T-A, A-T-C, G-C-C and G-T-A), significant association was observed in haplotype A-C-A (Pooled OR = 1.762, *P*-value  = 2.00E-03) and G-C-C (Pooled OR  = 0.649, *P*-value  = 2.00E-03) for Asians and the results were still significant after correcting for multiple comparison with a *P*-value of 0.012 (See Table 3 and Figure 2); however, no significant association was observed in G-C-C for all samples or Caucasian samples. Besides, no significant association was observed in other haplotypes (See Table 3).

### Sensitivity analysis

To determine whether a specific variable would impact the overall results, we compared results before and after removing studies with significant deviation from HWE (rs1800871 and rs1800872 in study [21] and rs1800896 in study [28]). The analysis showed no significant difference, which indicated that the results of the meta-analysis were not biased by studies with significant deviation from HWE (See Table S1).

### Heterogeneity and publication bias

Among three SNPs, significant heterogeneity was observed in rs1800896 with *P*-value <0.1. After stratifying for populations, no significant heterogeneity was observed in Asians, but the heterogeneity was still significant in Caucasians. Similarly, among six haplotypes, significant heterogeneity was observed in G-C-C with *P*-value <0.1; after stratifying for populations, no significant heterogeneity was observed in Asians, but the heterogeneity was still significant in Caucasians. For publication bias, no significant results were observed with all *P*-value >0.05 of Egger's test.Besides, funnel plots of SNPs and haplotypes did not show significant publication bias either. Results of heterogeneity and publication bias are shown in Table 3 and Figure S1–S4.

## Discussion

Results from the meta-analysis showed a significant association between rs1800872 of *IL-10* and risk of SZ, with an OR of 1.351 for genotype A/A, indicating a higher risk with SZ. As there exist difference in allele frequencies of rs1800872 among different ethnic groups (allele A frequency in cases/controls: 0.248∼0.279/0.212∼0.304 in Caucasians; 0.559∼0.719/0.502∼0.6986 in Asians), subgroup analysis was conducted. After stratifying for ethnicity, significant association was still observed under recessive model in both Caucasians (OR = 1.625, P-value  = 0.002) and Asians (OR = 1.264, P-value  = 0.013), indicating that the significance does not vary across different ethnic groups. Similarly, for rs1800896, no significant results were observed under any genetic model when all samples were included. Considering the difference in allele frequencies among different ethnic groups (allele G frequency in cases/controls: 0.345∼0.620/0.287∼0.504 in Caucasians; 0.035∼0.09/0.058∼0.084 in Asians), subgroup analysis was also conducted. After stratifying for ethnicity, significant results were observed in neither Caucasians nor Asians, suggesting these results did not vary across ethnic groups either. For rs1800871, no significant results were observed, as all included studies were Asian samples (allele C frequency in cases/controls: 0.29∼0.357/0.281∼0.379), the association in other enthic groups were worthy of being investigated further.

Besides, among six haplotypes of rs1800896-rs1800871-rs1800872, two haplotypes, A-C-A and G-C-C, were both observed to be significantly associated with SZ in Asians, with an OR of 1.762 and 0.649 respectively. For haplotype A-C-A (frequency in cases/controls: 0.013∼0.063/0.009∼0.048), A-T-A (frequency in cases/controls: 0.120∼0.688/0.113∼0.685) and A-T-C (frequency in cases/controls: 0.015∼0.145/0.016∼0.165), all included studies were Asian samples. The lack of Caucasian samples was due to the the absence of these haplotypes in Caucasians [39]. For haplotype A-C-C, although haplotype frequency varied across different enthic groups (frequency in cases/controls: 0.321∼0.335/0.341∼0.413 in Caucasians; 0.061∼0.246/0.065∼0.259 in Asians), stratification for ethnicity did not cause any change on the insignificant association with SZ. For haplotype G-C-C, haplotype frequency were also different among ethnic groups (frequency in cases/controls: 0.314∼0.401/0.411∼0.283 in Caucasians; 0.048∼0.012/0.060∼0.015 in Asians), subgroup analysis demonstrated significant association was only observed in Asian samples, indicating that the frequency difference between Caucasians and Asians might be a cause of different association with SZ between these two ethnic groups.

For rs1800872 (-592A/C), an SNP locating within a putative STAT-3 binding site and negative regulatory region of *IL-10*, it was reported that the C allele of this polymorphism correlates with higher *IL-10* production. Furthermore, it was also reported that carriers of haplotype G-C-C had higher *IL-10* production [40] – [41]. Combining with results from our meta-analysis, it is suggested that individuals with lower *IL-10* production genotypes might have a higher risk of SZ, compared with those with higher *IL-10* production genotypes. This was supported by a previous study demonstrating that excessive prenatal levels of *IL10* could decrease the risk of behavioral dysfunctions in the grown offspring [16]. In addition, clinical studies also reported anti-inflammatory drugs could improve the symptoms of SZ patients [14], as *IL-10* was a cytokine with anti-inflammatory effect [42]. This might be a possible explanation for the higher risk of SZ in carriers with lower *IL-10* production genotypes.

There are still some limitations for this study: 1) as limited statistical power is a common problem in genetic association studies, in our meta-analysis, negative results should be interpreted cautiously and still need to be further investigated in larger scale of samples. 2) In some cases, heterogeneity was not resolved after subgroup analyses, suggesting that other factors such as the differences in assays or clinical characteristics might have caused heterogeneity. 3). The lack of clinical information such as age onset of patients made us unable to further investigate the association of diseases with more detailed factors.

## Conclusion

As far as we know, this is the first meta-analysis to investigate the association between *IL-10* polymorphisms and risk of SZ. In this study, rs1800872 of three investigated SNPs of *IL-10* was observed to be significantly associated with SZ. Meanwhile, significant associations were also presented in haplotypes A-C-A and G-C-C among six haplotypes of rs1800896 - rs1800871 - rs1800872 for Asians, even after adjusting for multiple comparisons. The overall effect of this meta-analysis suggested that *IL-10* might be a risk factor of SZ. Larger and well-designed studies based on different ethnic groups are needed to confirm our results.

## Supporting Information

Figure S1 **Funnel plot of rs1800896.**
(DOC)

Figure S2 **Funnel plot of rs1800871.**
(DOC)

Figure S3 **Funnel plot of rs1800872.**
(DOC)

Figure S4 **Funnel plot of six haplotypes of rs1800896- rs1800871-rs1800872.**
(DOC)

Table S1 **Results of sensitivity analysis.**
(DOC)

Checklist S1 **PRISMA Checklist.**
(DOC)

Diagram S1 **PRISMA Flow Diagram.**
(DOC)
