# Supplementary figures and images for: Association of Interleukin-10 Polymorphisms with Schizophrenia: A Meta-Analysis
Source: PLoS One. 2014 Mar 6;9(3):e90407. doi: 10.1371/journal.pone.0090407 (PMC3946087; doi:10.1371/journal.pone.0090407)

**Figure S1 Funnel plot of rs1800896**


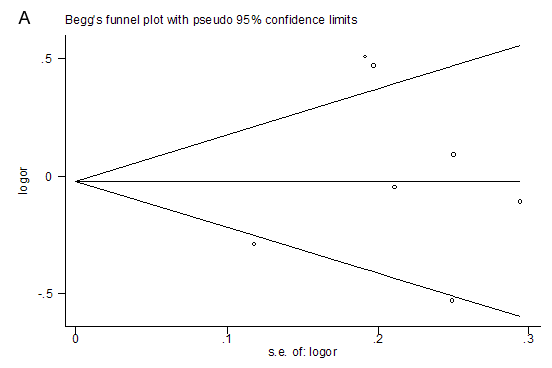

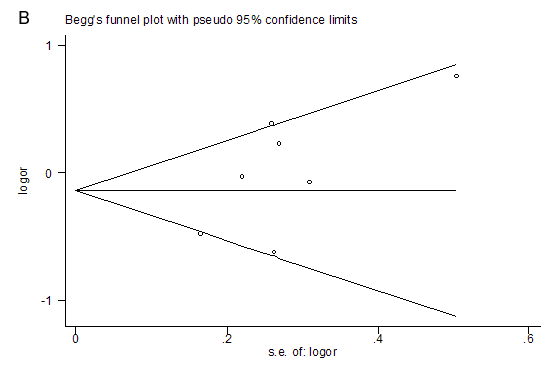

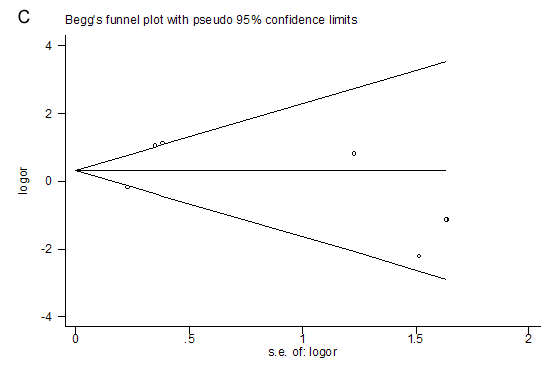


A: Allele G vs. allele A; B: GG+GA vs. AA; C: GG vs. GA+AA

Supplement: Figure S1 — Funnel plot of rs1800896. (DOC) [file pone.0090407.s001.doc]

**Figure S2 Funnel plot of rs1800871**


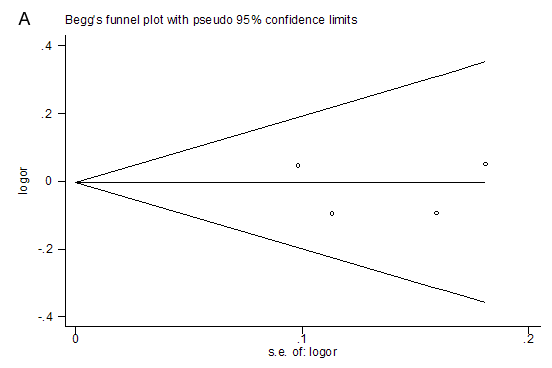

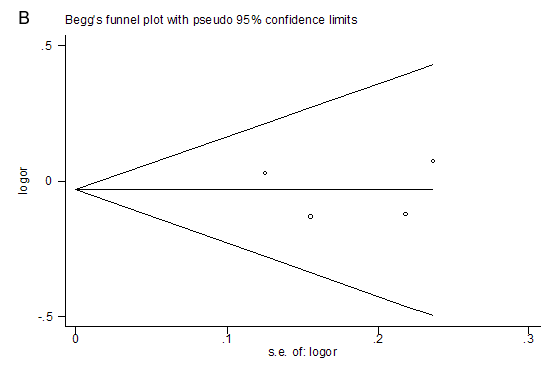

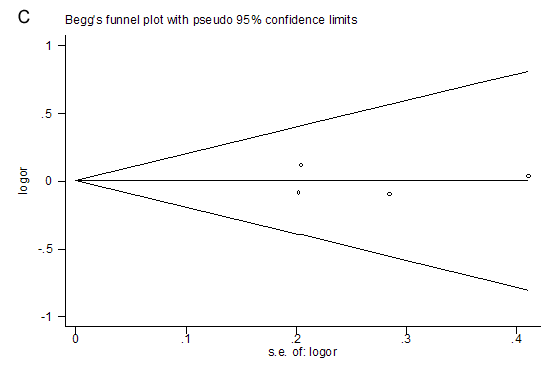


A: Allele C vs. allele T; B: CC+CT vs. TT; C: CC vs. CT+TT

Supplement: Figure S2 — Funnel plot of rs1800871. (DOC) [file pone.0090407.s002.doc]

**Figure S3 Funnel plot of rs1800872**


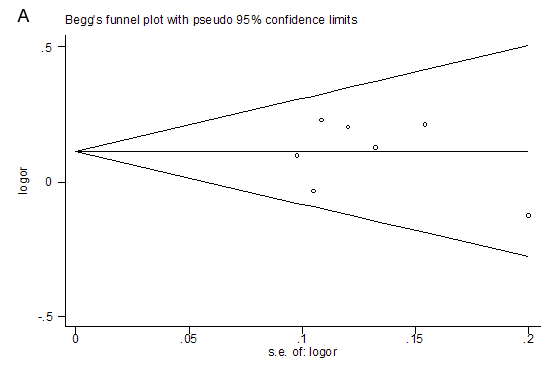


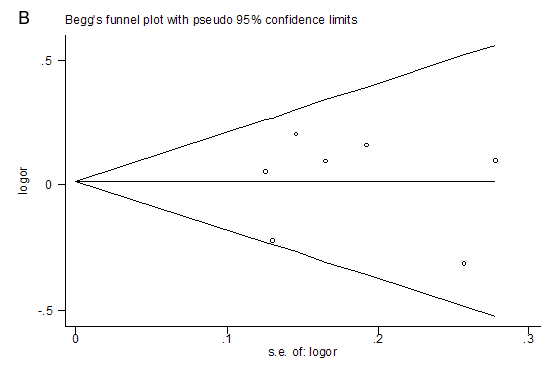


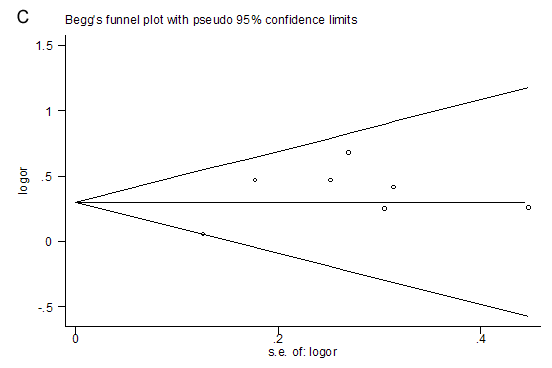


A: Allele A vs. allele C; B: AA+AC vs. CC; C: AA vs. AC+CC

Supplement: Figure S3 — Funnel plot of rs1800872. (DOC) [file pone.0090407.s003.doc]
