# Supplementary material for: Association of Interleukin-10 Polymorphisms with Schizophrenia: A Meta-Analysis
Source: PLoS One. 2014 Mar 6;9(3):e90407. doi: 10.1371/journal.pone.0090407 (PMC3946087; doi:10.1371/journal.pone.0090407)
Supplement: Figure S4 — Funnel plot of six haplotypes of rs1800896- rs1800871-rs1800872. (DOC) [file pone.0090407.s004.doc]

**Figure S4 Funnel plot of six haplotypes of rs1800896- rs1800871-rs1800872**


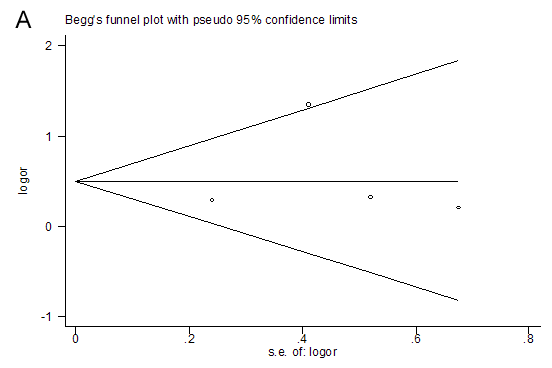

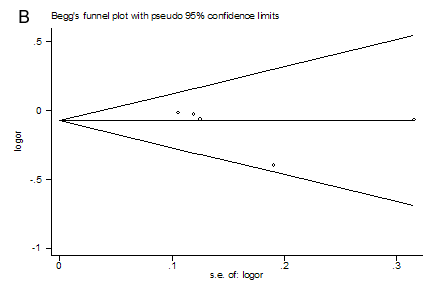

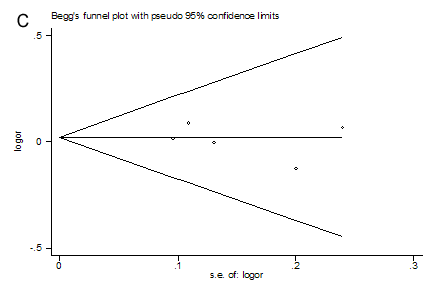

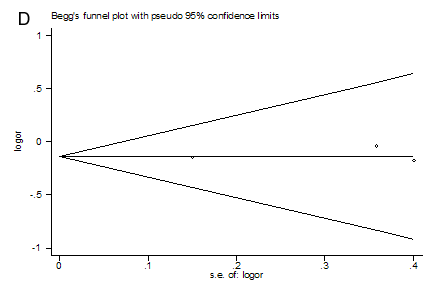

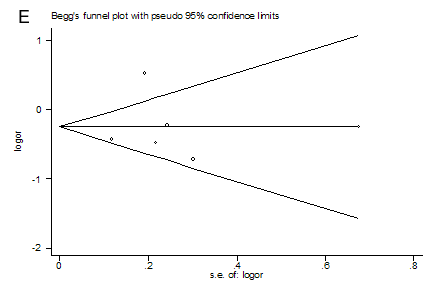

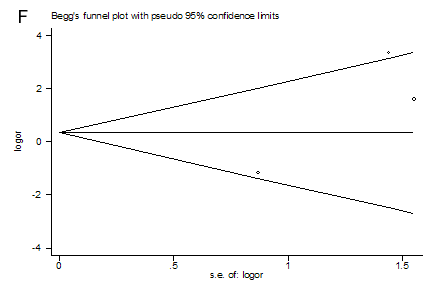


A: haplotype A-C-A; B: haplotype A-C-C; C: haplotype A-T-A; D: haplotype A-T-C; E: haplotype G-C-C; F: haplotype G-T-A
