# Supplementary material for: Association of Interleukin-10 Polymorphisms with Schizophrenia: A Meta-Analysis
Source: PLoS One. 2014 Mar 6;9(3):e90407. doi: 10.1371/journal.pone.0090407 (PMC3946087; doi:10.1371/journal.pone.0090407)
Supplement: Table S1 — Results of sensitivity analysis. (DOC) [file pone.0090407.s005.doc]

**Table S1 Results of sensitivity analysis**

| **SNP** | **Genetic model** | **Excluding studies with significant HWE** | |  | **Including studies with significant HWE** | |
| --- | --- | --- | --- | --- | --- | --- |
|  |  | **Pooled OR(95% CI)** | **P-value** |  | **Pooled OR(95% CI)** | **P-value** |
| rs1800096 | allele G vs. allele A | 0.943(0.700 - 1.271) | 0.702 |  | 1.022(0.758 - 1.377) | 0.888 |
|  | GG+GA vs. AA | 0.891(0.647 - 1.226) | 0.477 |  | 0.953(0.688 - 1.32) | 0.772 |
|  | GG vs. GA+AA | 1.049(0.419 - 2.629 ) | 0.918 |  | 1.351(0.621 - 2.942) | 0.448 |
| rs1800872 | allele A vs. allele C | 1.091(0.988 - 1.205) | 0.084 |  | 1.12(1.023 - 1.225) | 0.126 |
|  | AA+AC vs. CC | 0.999(0.880 - 1.136) | 0.994 |  | 1.016(0.9 - 1.147) | 0.796 |
|  | AA vs. AC+CC | 1.291(1.080 - 1.544) | 5.00E-03 |  | 1.351(1.153 - 1.584) | 1.86E-03 |
| rs1800871 | allele C vs. allele T | 1.015(0.875 - 1.177) | 0.847 |  | 0.98(0.867 - 1.109) | 0.752 |
|  | CC+CT vs. TT | 1.039 (0.809 -1.334) | 0.766 |  | 0.967(0.822 - 1.139) | 0.688 |
|  | CC vs. CT+TT | 0.982 (0.667 - 1.445) | 0.927 |  | 0.997(0.785 - 1.267) | 0.98 |
