# Supplementary material for: Association of Interleukin-10 Polymorphisms with Schizophrenia: A Meta-Analysis
Source: PLoS One. 2014 Mar 6;9(3):e90407. doi: 10.1371/journal.pone.0090407 (PMC3946087; doi:10.1371/journal.pone.0090407)
Supplement: Checklist S1 — PRISMA Checklist. (DOC) [file pone.0090407.s006.doc]

| **Section/topic** | **#** | **Checklist item** | **Reported on page #** |
| --- | --- | --- | --- |
| **TITLE** | | |  |
| Title | 1 | Association of interleukin-10 polymorphisms with schizophrenia: a meta-analysis | Title section |
| **ABSTRACT** | | |  |
| Structured summary | 2 | **Background:** The involvement of cytokines in schizophrenia (SZ) has been proposed in recent years and various studies have accumulated convergent lines of evidence. Among which, the role of interleukin-10 (IL-10) in SZ has been explored in a number of studies by investigating association of single nucleotide polymorphisms (SNPs) and susceptibility of SZ. However, the results are inconsistent since its power is limited by the individual sample size. To evaluate the overall effect between them, we conducted a meta-analysis by combining all available studies.  **Methods:** Studies were searched from the database of PubMed, PsycINFO and ISI web of Knowledge up to Nov 2013. The meta-analysis was conducted based on statement of preferred reporting items for systematic reviews and meta-analyses (PRISMA).  **Results:** Eleven studies including 6399 subjects (3129 cases and 3270 controls) were available for the meta-analysis. Among three investigated SNPs, rs1800872 was observed to be significantly associated with risk of SZ (AA vs. AC + CC, Pooled OR = 1.351, P-value = 2.06E-04). Meanwhile, among six haplotypes of rs1800896 - rs1800871 - rs1800872, significant associations were observed in haplotype A-C-A (Pooled OR = 1.762, P-value = 2.00E-03) and G-C-C (Pooled OR = 0.649, P-value = 2.00E-03) for Asians. These results were still significant after adjusting for multiple comparisons.  **Conclusions:** This meta-analysis demonstrated an SNP and two haplotypes of IL-10 significantly associated with SZ, suggesting that IL-10 might be a risk factor of SZ. | Abstract  section |
| **INTRODUCTION** | | |  |
| Rationale | 3 | During these decades, researches from different areas have provided convergent lines of evidence for the involvement of cytokines in SZ. With the emergence of the evidence, abundant genetic researches have been conducted to explore the genetic basis of this hypothesis. Among these cytokines, the role of interleukin-10 (IL-10) in SZ has been explored in a number of studies by investigating association of single nucleotide polymorphisms (SNPs) and susceptibility of SZ. However, results of individual studies were inconsistent and might not be powerful enough due to the limited sample size. | Introduction section, Paragraph 1-3 |
| Objectives | 4 | To evaluate the overall effect of IL-10 polymorphisms on SZ, a meta-analysis was conducted in the present study by pooling all available data together. | Introduction section, Paragraph 4 |
| **METHODS** | | |  |
| Protocol and registration | 5 | Not applicable | - |
| Eligibility criteria | 6 | Publication date and publication language were not limited in our search. In addition, we also examined manually the references and supplements of relevant studies to find additional studies. If the information is insufficient for meta-analysis, we requested data from the corresponding author. Besides, If overlapped samples were used in different studies, we excluded overlapping samples or keep the study with the largest sample size. | “Search Strategy” of “Materials and Methods” section |
| Information sources | 7 | The database of PubMed, PsycINFO and ISI web of Knowledge were searched up to November 2013. In addition, we also examined manually the references and supplements of relevant studies to find additional studies. Meanwhile, published genome-wide association studies (GWASs) about schizophrenia were also examined. | “Search Strategy” of “Materials and Methods” section |
| Search | 8 | Search terms in PubMed,: ("interleukin 10"[all fields] OR "interleukin-10"[all fields] OR "IL10"[all fields] OR "IL 10"[all fields] OR "IL-10"[all fields]) AND "Schizophrenia"[all fields] | “Search Strategy” of “Materials and Methods” section |
| Study selection | 9 | Studies aiming to examine the association between IL-10 polymorphisms and susceptibility of SZ were included. Moreover, studies had to fulfill all of the following criteria: 1) a case-control design comparing patients with SZ to controls without mental disorders; 2) patients were diagnosed with well-validated diagnostic criteria (e.g. The Diagnostic and Statistical Manual of Mental Disorders); 3) controls were free of autoimmune or inflammatory diseases; 4) original data of genotype frequencies were published or provided by the authors. Studies were excluded if one of the following existed: 1) studies used family-based or cohort design; 2) samples were cases only; 3) genotype frequencies were neither published nor provided; 4) information is still insufficient for the meta-analysis even after requesting from authors. | “Selection Criteria” of “Materials and Methods” section |
| Data collection process | 10 | All data were extracted independently by two authors according to the inclusion criteria listed above. Disagreements were resolved by discussion between the two authors. The following characteristics were collected from each study: the first author, publication year, geographic region, ethnicity, diagnostic criteria, gender component, sample size, age of cases, age of controls, SNPs/haplotypes investigated, and distribution of genotypes among cases and controls for each involved SNP/haplotype. | “Data Extraction” of “Materials and Methods” section |
| Data items | 11 | Not applicable | - |
| Risk of bias in individual studies | 12 | As deviations from HWE in control subjects may bias the estimates of genetic effects in a meta-analysis [30], sensitivity analysis was conducted to examine such influence by removing studies with significant deviation from HWE in control subjects and recalculating the pooled OR and 95% CI. | Paragraph 3 in “Data analysis” of “Materials and Methods” section |
| Summary measures | 13 | The strength of association was expressed as pooled odds ratio (OR) along with the corresponding 95% confidence interval (CI), which were estimated for each study in a random-effects model or in a fixed-effects model. If there was a significant heterogeneity (P-value < 0.1), a random-effects model (the DerSimonian and Laird method) was selected to pool the data. Otherwise, a fixed-effects model (the Mantel-Haenszel method) was selected to pool the data. As suggested in previous studies, for each polymorphism, pooled ORs were calculated under the following genetic models: additive model (allele a vs. allele A), dominant model (a/a + A/a vs. A/A), recessive model (a/a vs. A/a + A/A), in which “a” represented the minor allele and “A” represented the major allele. The significance of pooled ORs was determined by Z-test and P-value < 0.05 was considered as statistically significant. Moreover, corrections for multiple comparisons were conducted by the Bonferroni method. | Paragraph 1 and 2 in “Data analysis” of “Materials and Methods” section |
| Synthesis of results | 14 | Heterogeneity among studies was systematically calculated with the χ^2^ -based Q testing and I^2^ statistics. P-value < 0.1 was considered as significant and I^2^ was interpreted as the proportion of total variation contributed by between-study variation. Heterogeneity was also quantified using the I^2^ metric (I^2^ < 25%, no heterogeneity; I^2^ = 25–50%, moderate heterogeneity; I^2^ > 50%, large or extreme heterogeneity). | Paragraph 4 in “Data analysis” of “Materials and Methods” section |

Page 1 of 2

| **Section/topic** | **#** | **Checklist item** | **Reported on page #** |
| --- | --- | --- | --- |
| Risk of bias across studies | 15 | Publication bias was examined using the Egger’s tests with funnel plots. If there is evidence of publication bias, the funnel plot is noticeably asymmetric. For the Egger’s tests the significance level was set at 0.05. | Paragraph 4 in “Data analysis” of “Materials and Methods” section |
| Additional analyses | 16 | Subgroup analyses were also conducted to assess any moderating effects of ethnicity (Caucasian and Asian) on odds ratios derived from each study if significant heterogeneity was observed in the meta-analysis. | Paragraph 2 in “Data analysis” of “Materials and Methods” section |
| **RESULTS** | | |  |
| Study selection | 17 | A total of 63 papers were obtained with the initial search of databases. After screening, ten studies fulfilled the inclusion criteria, from which genotype data of three SNPs of IL-10 were obtained. Furthermore, one dataset of genotype frequencies of one SNP (rs1800872) were also acquired from a genome-wide association study of schizophrenia (See Figure1). | Paragraph 1 of “Results” section |
| Study characteristics | 18 | Combining data of candidate gene association study with GWAS data, eleven studies with a total of 6399 participants (3129 cases and 3270 controls) were available for this meta-analysis (shown in Table 1). The qualities of these studies were considered accessible for the meta-analysis. The flow chart of selection of studies and reasons for exclusion are presented in Figure 1. Data of three IL-10 SNPs (rs1800896, rs1800871 and rs1800872) and six haplotypes of rs1800896-rs1800871-rs1800872 were meta-analyzed (shown in Table 2). Characteristics of studies and genotype frequencies were presented in Tables 1 and 2 respectively. | Paragraph 1 of “Results” section |
| Risk of bias within studies | 19 | The analysis showed no significant difference, which indicated that the results of the meta-analysis were not biased by studies with significant deviation from HWE (See Table S1). | Table S1 |
| Results of individual studies | 20 | Results of meta-analysis are shown in Table 3. | Table 3 |
| Synthesis of results | 21 | Results of meta-analysis are shown in Table 3. | Table 3 |
| Risk of bias across studies | 22 | For publication bias, no significant results were observed with all P-value > 0.05 of Egger’s test. Results of heterogeneity and publication bias are shown in Table 3 and Figure S1-S4. | Table 3, Figure S1-S4 |
| Additional analysis | 23 | Among three SNPs, significant heterogeneity was observed in rs1800896 with P-value < 0.1. After stratifying for populations, no significant heterogeneity was observed in Asians, but the heterogeneity was still significant in Caucasians. Similarly, among six haplotypes, significant heterogeneity was observed in G-C-C with P-value < 0.1; after stratifying for populations, no significant heterogeneity was observed in Asians, but the heterogeneity was still significant in Caucasians. | Table 3 |
| **DISCUSSION** | | |  |
| Summary of evidence | 24 | Among three investigated SNPs, rs1800872 was observed to be significantly associated with risk of SZ (AA vs. AC + CC, Pooled OR = 1.351, P-value = 2.06E-04). Meanwhile, among six haplotypes of rs1800896 - rs1800871 - rs1800872, significant associations were observed in haplotype A-C-A (Pooled OR = 1.762, P-value = 2.00E-03) and G-C-C (Pooled OR = 0.649, P-value = 2.00E-03) for Asians. These results were still significant after adjusting for multiple comparisons. | Paragraph 1 of “Discussion” section |
| Limitations | 25 | 1) as limited statistical power is a common problem in genetic association studies, in our meta-analysis, negative results should be interpreted cautiously and still need to be further investigated in larger scale of samples. 2) In some cases, heterogeneity was not resolved after subgroup analyses, suggesting that other factors such as the differences in assays or clinical characteristics might have caused heterogeneity. 3). The lack of clinical information such as age onset of patients made us unable to further investigate the association of diseases with more detailed factors. | Paragraph 4 of “Discussion” section |
| Conclusions | 26 | As far as we know, this is the first meta-analysis to investigate the association between IL-10 polymorphisms and risk of SZ. In this study, rs1800872 of three investigated SNPs of IL-10 was observed to be significantly associated with SZ. Meanwhile, significant associations were also presented in haplotypes A-C-A and G-C-C among six haplotypes of rs1800896 - rs1800871 - rs1800872 for Asians, even after adjusting for multiple comparisons. The overall effect of this meta-anlaysis suggested that IL-10 might be a risk factor of SZ. Larger and well-designed studies based on different ethnic groups are needed to confirm our results. | “Conclusion” section |
| **FUNDING** | | |  |
| Funding | 27 | This research was supported by the Knowledge Innovation Program of the Chinese Academy of Sciences (KSCX2-EW-J-8), the CAS/SAFEA International Partnership Program for Creative Research Teams (Y2CX131003), the Strategic Priority Research Program (B) of the Chinese Academy of Sciences (XDB02030002) and Key Laboratory of Mental Health, Institute of Psychology, Chinese Academy of Sciences. | - |

*From:*  Moher D, Liberati A, Tetzlaff J, Altman DG, The PRISMA Group (2009). Preferred Reporting Items for Systematic Reviews and Meta-Analyses: The PRISMA Statement. PLoS Med 6(6): e1000097. doi:10.1371/journal.pmed1000097

For more information, visit: **www.prisma-statement.org**.

Page 2 of 2
