# Supplementary material for: Association of Interleukin-10 Polymorphisms with Schizophrenia: A Meta-Analysis
Source: PLoS One. 2014 Mar 6;9(3):e90407. doi: 10.1371/journal.pone.0090407 (PMC3946087; doi:10.1371/journal.pone.0090407)
Supplement: Diagram S1 — PRISMA Flow Diagram. (DOC) [file pone.0090407.s007.doc]

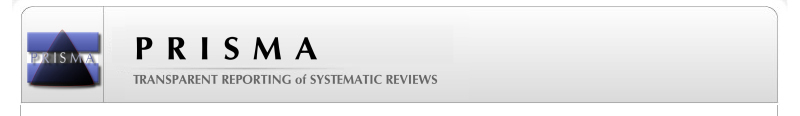
**PRISMA 2009 Flow Diagram**

**Screening**

**Included**

**Eligibility**

**Identification**

Records identified through database searching
(n = 63)

Additional records identified through other sources
(n =1)

Records after duplicates removed
(n = 64)

Records screened
(n = 19)

Records excluded
(n =45 )

Full-text articles assessed for eligibility
(n = 14)

Full-text articles excluded, with reasons
(n =3 )

Studies included in qualitative synthesis
(n = 11)

Studies included in quantitative synthesis (meta-analysis)
(n =11)
